# Supplementary material for: Identification of photoperiod-induced specific miRNAs in the adrenal glands of Sunite sheep (Ovis aries)
Source: Front Vet Sci. 2022 Jul 22;9:888207. doi: 10.3389/fvets.2022.888207 (PMC9354845; doi:10.3389/fvets.2022.888207)
Supplement: Supplementary file 1 [file Data_Sheet_1.ZIP › Supplementary file/miRDeep2 file.zip!/miRDeep2 file/Novel_Structure/SPLP42A2/Novel_537.pdf]

Provisional ID : NC_019482.2_41145
Score total : 1.7
Score for star read(s) : -1.3
Score for read counts : 0
Score for mfe : 1.4
Score for randfold : 1.6
Score for cons. seed :
Total read count : 43
Mature read count : 43
Loop read count : 0
Star read count : 0

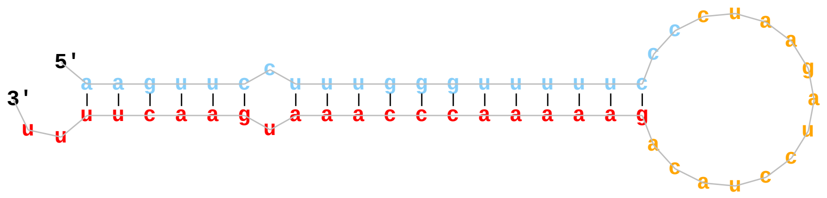

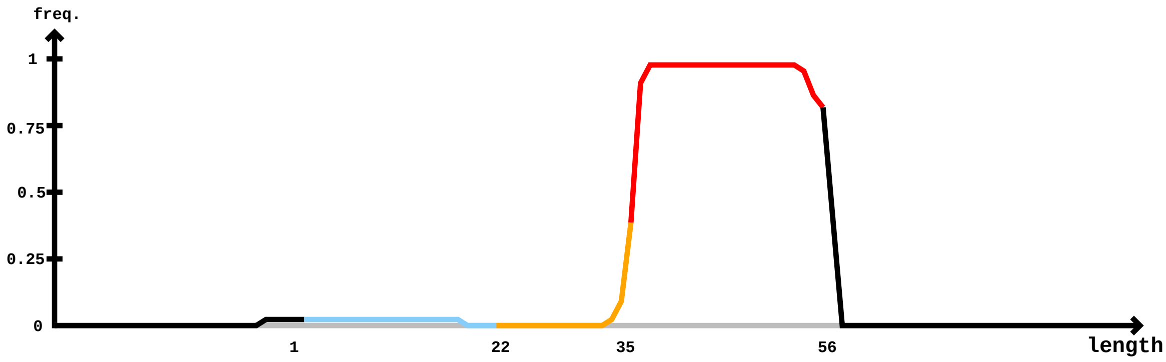

Star Mature

```
5'- ccuuuuaguauuggguuagcuaaaaaguuccuuuggguuuuuccccuaagauccuacagaaaaacccaaaugaacuuuugggccaaccuaaaugaaugaaaaugguuugug   -3'    exp
    (((((((...(((((((.(((.((((((((.(((((((((((((.............))))))))))))).)))))))).))).))))))).....)))))..))......    reads      mm        sample
    ....................uaaaaaguuccuuuggguuuu......................................................................    1          0         seq
    ........................................................cagaaaaacccaaaugaacuuu.................................    1          0         seq
    .........................................................Ugaaaaacccaaaugaacuu..................................    1          1         seq
    .........................................................agaaaaacccaaaugaacuuu.................................    1          0         seq
    .........................................................agaaaaacccaGaugaacuuuu................................    1          1         seq
    ..........................................................gaaaaacccaGaugaacuu..................................    3          1         seq
    ..........................................................gaaaaacccaGaugaacuuuu................................    10         1         seq
    ...........................................................aaaaacccaGaugaacu...................................    1          1         seq
    ...........................................................aaaaacccaaaugaacuuuu................................    3          0         seq
    ...........................................................aCaaacccaaaugaacuuuu................................    1          1         seq
    ...........................................................aaaaacccaGaugaacuuuu................................    5          1         seq
    ...........................................................aaaaacccaaaugaacuuuuC...............................    1          1         seq
    ...........................................................aaaaacccGaaugaacuuuug...............................    10         1         seq
    ...........................................................aaaaacccaaaugaacuuuuA...............................    1          1         seq
    ...........................................................aCaaacccaaaugaacuuuug...............................    1          1         seq
    ............................................................aaaacccGaaugaacuuuug...............................    2          1         seq
    ............................................................aaaacccaaaugaacuuuuC...............................    1          1         seq
```
